# Production Phase Affects the Bioaerosol Microbial Composition and Functional Potential in Swine Confinement Buildings

**DOI:** 10.3390/ani9030090

**Published:** 2019-03-12

**Authors:** Honglin Yan, Li Zhang, Zhendong Guo, Hongfu Zhang, Jingbo Liu

**Affiliations:** 1 School of Life Science and Engineering, Southwest University of Science and Technology, Mianyang 621010, China; honglinyan@swust.edu.cn (H.Y.); faswust@126.com (L.Z.); 2 State Key Laboratory of Animal Nutrition, Institute of Animal Sciences, Chinese Academy of Agricultural Sciences, Beijing 100000, China; zhanghfcaas@gmail.com; 3 Key Labortatory of Jilin Province for Zoonosis Prevention and Control, Veterinary Research Institute, Chinese Academy of Agriculture Sciences, Changchun 130122, China; guozd@foxmail.com

**Keywords:** production phase, microbiome, swine confinement buildings, bioaerosol

## Abstract

**Simple Summary:**

In the present study, air samples from different types of swine confinement buildings (SCBs), which exclusively housed weaning piglets (WP), finishing pigs (FP), farrowing sows (FS), gestating sows (GS), and breeding boars (BB), respectively, were used to study the effects of the production phase on the taxonomical composition and functional potential of microbial communities in the SCBs bioaerosols (airborne particles that are biological in origin). Whole metagenome shotgun sequencing, which is the untargeted (‘shotgun’) sequencing of all microbial genomes (‘metagenome’) present in a sample, was adopted to profile the bioaerosol microbiome (full collection of genes of all the microbes in a community). The results showed that bioaerosol microbiome of BB shared a high similarity with GS, and WP bioaerosol microbiome was more similar to FP than other types of SCBs. The findings of this study suggested that the production phase of pigs contributes to the variations of SCBs bioaerosol microbiome.

**Abstract:**

Bioaerosols from swine confinement buildings (SCBs) pose a challenge to public health, and microorganisms within the SCBs bioaerosols originate from swine feces, of which the microbial composition is associated with the production phase. The present study adopted the whole metagenome shotgun sequencing approach, to assess the effects of the production phase on the composition and functional potential of microbial populations in SCBs bioaerosols. Most annotated proteins were assigned into domain bacteria, within which the predominant phylum was *Firmicutes*. The taxonomical profiles of bioaerosols from different types of piggeries showed that buildings housing weaning piglets (WP) exhibited higher abundances of *Bacteroidetes* and *Proteobacteria* than buildings housing finishing pigs (FP), gestating sows (GS), farrowing sows (FS), and breeding boars (BB). Regarding the functional potential, the WP bioaerosol had more genes involved in the protein turnover and fewer genes involved in the carbohydrate metabolism than bioaerosols from other types of SCBs. Furthermore, production phase influenced the antibiotic resistance genes (ARGs) profile of the SCBs bioaerosols. Bioaerosol microbiome of BB, shared a high similarity with GS, and WP bioaerosol microbiome was more similar to FP than other types of SCBs. Our study suggests that the production phase plays a key role in the SCBs bioaerosol microbiome.

## 1. Introduction

In recent years, swine production has been industrialized all over the world, resulting in an increase in the use of confined buildings. The high densities of animals raised in such confined areas can create a poor indoor air quality [1]. Bioaerosols in the swine confinement buildings (SCBs), consisted of airborne microorganisms, their constituent parts, and by-products, have been shown to cause diverse respiratory diseases or symptoms, like allergic asthma and airway inflammation, in both farm workers and animals [2,3]. Moreover, because of their small size and light weight, bioaerosols can be easily emitted into the external environment as a result of intensive farming, and pose a significant challenge to public health [4].

Microbial aerosols, including bacteria and fungi, have been recognized as the main contributors to the adverse health outcomes of SCBs bioaerosols, and thus, characterization of the microbial community of SCBs bioaerosols has drawn much attention of the aerobiology researchers [4,5]. The composition of the SCBs bioaerosol microflora, depend primarily on the fecal microbiota of pigs, within the stable, as most of microorganisms in SCBs bioaerosols originate from swine manure [6]. Likewise, Kristiansen et al. applied the molecular biology method to examine the diversity and abundance of bacteria and fungi in SCBs bioaerosols and have suggested that the bioaerosol populations share a high similarity with the fecal microbiota of confined pigs [7]. A more recent study using next-generation sequencing methods have further proven that the major source of microbial bioaerosols in SCBs, is swine feces [8]. Growing evidence suggests that the swine gut microbiota changes with the growth phase, as indicated by the gradual replacement of *Bacteroidetes* by *Firmicutes* at the phylum level and the continuous decline of *Prevotella* at the genus level, from birth to marketing [9,10,11]. Therefore, it can be inferred that bioaerosols from different types of piggeries, which exclusively house pigs at different growth periods, might harbor distinct microbiota composition. Hong et al. proved this speculation, showing that the taxonomical profile of bioaerosols from gestation and farrowing piggeries were more similar to each other than to the weaning and finishing piggeries [2]. However, the microbial functional capabilities and the occurrence and diversity of antibiotic resistant genes (ARGs) in bioaerosols from different types of piggeries remains to be investigated. The functional capabilities and ARGs profile of a microbial community have been associated with its taxonomical profile [12]. Thus, we hypothesized that the production phase would affect the microbial functional potential and ARGs profile of the aerosols in the SCBs.

Previous studies investigating the bioaerosol microbiota in SCBs have been performed, predominantly, by using culture-based techniques [13,14,15], and only a few studies have estimated the bioaerosol microbial composition in SCBs by culture-independent approaches, like sequence technologies targeting sub-regions of the 16S rRNA gene [2,6,7]. Most of these mentioned methods are limited in scope. Culture-dependent methods contain an inherent bias, as most microorganisms are not easily culturable [16]. The 16S amplicon sequencing method is based on the putative association of the 16S rRNA gene, with operational taxonomic units (OTUs), it cannot directly identify functional capabilities and the ARGs profile of the microbes under study [17]. However, whole metagenome shotgun sequencing (WMS) aims to sample all genes from a community and can provide detailed metabolic and functional profiles [5,18,19]. Thus, the WMS technique was adopted in the present study, to determine the microbial composition, functional potential, and ARGs profile of the bioaerosols sampled from SCBs. In China and Singapore, swine production is generally classified into several phases, including gestation, farrowing, nursery, finishing, and breeding (referring to replacement gilts, sows, and boars, hereafter, and especially referring to the breeding boars). Pigs within each production phase are raised in a separate building [20]. Accordingly, the present study was designed to compare the microbial composition, functional potential, and ARGs profile in the bioaerosols, among the above-mentioned five production phases.

## 2. Materials and Methods

### 2.1. Sampling Sites

Samples were collected from a large-scale, modern swine farm of the New Hope Group in the Guizhou province, China. Within this swine farm, SCBs were classified into five categories, namely nurseries, finishing barn, gestation barn, farrowing barn, and boar barn, which exclusively housed weaning piglets (WP), finishing pigs (FP), gestating sows (GS), farrowing sows (FS), and breeding boars (BB). In both, the nurseries and the finishing piggeries, pigs were group housed in pens [21]; in the rest of the categories of piggeries, pigs were housed in individual stalls. WP and FP buildings were located in the fattening zone, and the GS, FS, and BB buildings were located in the breeding zone of the swine farm. The fattening zone was walled-off from the breeding zone. Piglets (Duroc × Landrace × Yorkshire) at 28 days of age in the breeding zone were transferred into the WP of the fattening zone for nursing. At the end of nursery phase, piglets were transferred into the FP for fattening, until market weight. SCBs were sterilized with a disinfectant, before transferring the pigs into the barn. The number of animals kept in the WP, FP, GS, FS, and the BB, ranged from 24 to 480, and the stocking density varied from 0.6 to 12 m^2^/head [22]. All SCBs were equipped with a mechanical ventilation system that was used to control the indoor temperature and humidity. The manure system employed in these SCBs was a deep manure pit, under a fully slatted floor. The light schedule for the five types of piggeries was 24 L. Pigs were fed with the feeds of the corresponding productions phase from the mills installed inside the farm. There was no history of porcine reproductive and respiratory syndrome virus (PRRSV), porcine epidemic diarrhea virus (PEDV) and other virus infections in this farm.

### 2.2. Sample Collection

**Figure 1:**
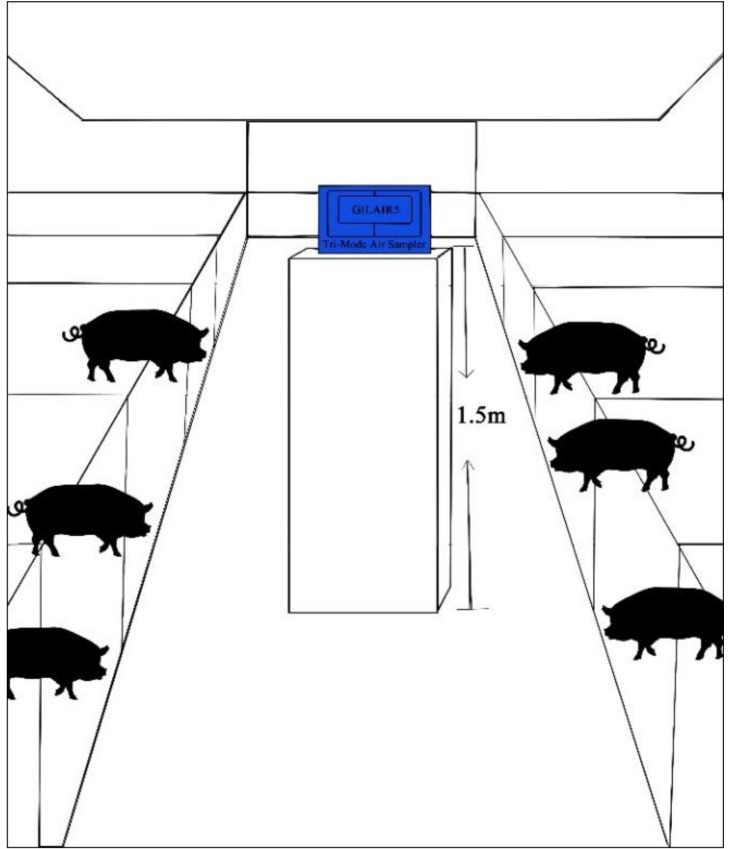
The location of air sampler in the swine confinement buildings.

In June of 2017, three SCBs from each production phase were chosen to collect the airborne samples. In each building, airborne sampling was done with a 37 mm glass fiber filter loaded onto the closed-face cassettes (Sunkyong Chemicals Ltd, SKC Ltd, Seoul, Korea) for 4 h, at a flow rate of 2 L/min. The 37 mm cassettes (SKC Ltd, Seoul, Korea) were connected to low-volume pumps (Gilian GilAir5 Tri-Mode Air Sampler, Sensidyne, LP, St. Petersburg, USA) (producer, city country) that had been calibrated with DryCal DC-2 flowmeter (Bios). The sampling devices were placed in the midpoint of the corridor, outside the pens, at a height of 1.5 m above the ground (Figure 1). The mass concentrations of the total suspended particles in the tested SCBs, ranged from 20.2 μg/m^3^ to 193.5 μg/m^3^. Air temperature and relative humidity were measured with a hygrothermograph (Qingsheng Electronic Technology Ltd., Handan, China). The air temperature in the WP, FP, GS, FS, and the BB was 27, 20, 20, 22, and 20 °C, respectively. The air humidity in the five types of piggeries ranged from 60% to 70%. The air speed in the SCBs was measured using an anemometer (model 6004, KANOMAX, Osaka, Japan). The air exchange rate in the five types of piggeries ranged from 0.25 m/s to 0.46 m/s. Following sampling, all cassettes were kept on ice and transported to the laboratory. Glass fiber filters from the SCBs, within the same category, were pooled in the sterilized ultrapure water, resulting in one pooled sample for each type of piggery. These were then vortexed vigorously at room temperature, for 20 min, to dissolve the aerosol particles. The resulting solutions were centrifuged at a speed of 21,000× *g* at 4 °C, for 10 min, and the pellets were stored at −20 °C, until the extraction of the metagenomic DNA.

### 2.3. Bioaerosol DNA Extraction and Metagenomic Library Preparation

The total genomic DNA of pellets was extracted using the UltraClean^®^ Soil DNA Isolation Kit (Mo Bio Laboratories, Carlsbad, CA, USA), according to the manufacturer’s instructions. The purified DNA was resuspended in 200 μL of DNase/RNase-free water and kept at −20 °C, until their use for sequencing. The concentration and quality of total DNA were assessed using NanoDrop ND-1000 (Nanodrop Technologies, Thermo Scientific, Wilmington, DE, USA) and agarose gel electrophoresis. Sterilized water samples were used as a negative control for DNA extraction, and the PCR results showed that the negative control water samples did not yield detectable 16S rRNA products.

For construction of the shotgun library, approximately 5 μg of the DNA sample was mechanically sheared to 350 base-pair fragments, using a Covaris S220 instrument (Covaris, Woburn, MA, USA). Libraries were constructed using the Apollo 324 Next Generation Library Preparation System (IntegenX, Pleasanton, CA, USA) with the NEXTflex-96 DNA barcodes (Bioo Scientific, Austin, TX, USA).

### 2.4. Metagenomic Sequencing and Read Assembly

The DNA libraries were sequenced on the HiSeq 2000 platform, by Novegene (Beijing, China). A PE101+8+101 cycle (Paired-end sequencing, 101-bp reads and 8-bp index sequence) sequencing strategy was adopted for the Illumina high-throughput sequencing. Raw data were filtered by removing the reads which contained more than three ambiguous nucleotides, had a length less than 35 bp, had an overlapping region with an adapter more than 15 bp, or had more than 40 nucleotides, with a quality value lower than 38. After quality filtering, the resulting clean data were assembled into contigs, using the SOAPdenovo software (V 2.04, with a setting of -d 1 (remove low frequency K-mer with frequency less than 1), -M 3 (the strength of merging similar sequences during contig is set as 3), -R (use reads to solve tiny repeats), -u (un-mask high coverage contigs before scaffolding), -F (use reads to fill the intra-scaffold gap), -K 55 (K-mer size is 55)) [23], and contigs longer than 500 bp were retained for downstream analysis. The contigs were used to predict the open reading frames (ORFs) with the METAGENEMARK software (V 2.10, with default settings) [24]. The ORFs that were longer than 100 nucleotides were then imported into the CD-HIT software (V 4.58, http://www.bioinformatics.org/cd-hit) [25], to remove the redundant sequences and determine the gene abundance and statistics among the samples; the parameter options were -c 0.95, -G 0, -aS 0.9, -g 1, -d 0 [26]. The clean data of each sample were mapped back to the non-redundant ORF sets, using SoapAligner (soap 2.21, with the setting of -m 200, -x 400, identity ≥ 95%) [27], and the coverage for each ORF was calculated as the number of mapped reads. The final non-redundant gene sets were generated from the non-redundant ORF sets, by filtering the genes which contained less than 2 mapped reads.

### 2.5. Taxonomical Prediction and Functional Annotation

The gene sets for all samples were annotated for phylogenetic and functional analysis, using the DIAMOND software (V 0.7.9) [28]. The protein sequences of functional genes were compared to the NCBI NR database (Version: 20161115, https://www.ncbi.nlm.nih.gov/), using BLASTP, with an e-value < 1 × 10^−5^. Sequences were assigned to NCBI taxonomies, using the MEGAN software [29], with the lowest common ancestor algorithm and the default parameters. For the metagenomic function annotation, the protein sequences of functional genes were subjected to a BLASTP search, against the KEGG database (version 59) [30] and the eggNOG database (version 3.0) [31], with an e-value < 1 × 10^−5^. The abundance of each KEGG Orthology (KO) in a sample was calculated from raw counts, and KEGG functional categories in each sample were generated by summing individual KO abundances. NOGs were processed in a similar fashion. To identify ARGs, the protein sequences of functional genes from each sample were subjected to a BLASTP, against the Antibiotic Resistance Database (ARDB, http://ardb.cbcb.umd.edu/), with an e-value < 1 × 10^−5^. The protein sequence with its best hit in the ARDB matched with a ≥90% amino acid identity over 25 amino acids, was annotated as an ARG-like sequence. The similarity among the taxonomical and functional profiles in five pooled bioaerosol samples was determined using either the principle coordinate analysis (PCoA) or the UPGMA cluster analysis (CA), based on Bray Curtis dissimilarity. PCoA was based on the abundances of genus and species, the KEGG profile, and the eggNOGs profile, and CA, based on the abundances of genus and species, were performed using R studio v3.4.1.

### 2.6. Data Deposition

The raw sequence data for all samples are available at NCBI, under the SRA database with the accession number PRJNA492489.

## 3. Results

### 3.1. Taxonomical Characterization of the Metagenomic Profiles

**Figure 2:**
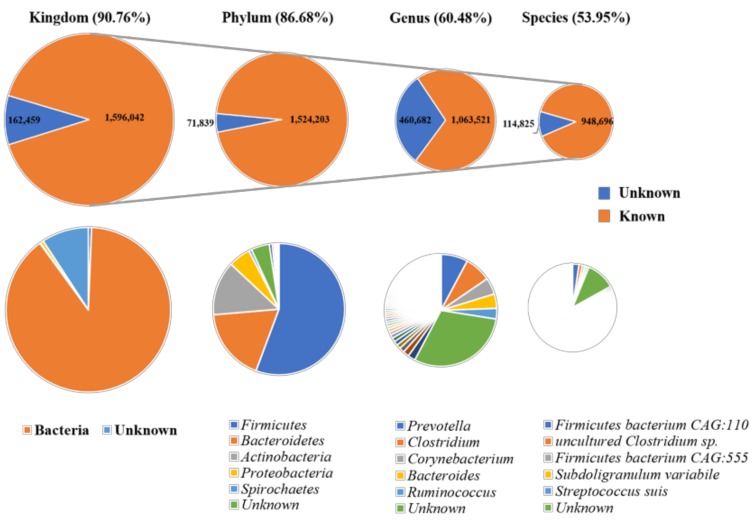
Taxonomical annotation of the bioaerosol catalogue to the kingdom, phylum, genus, and species levels.

We collected bioaerosol samples from 5 SCBs, which exclusively housed weaning piglets, finishing pigs, gestating sows, farrowing sows, and breeding boars, respectively. In total, we obtained 25.1 GB high-quality data, with an average of 5.02 GB per sample. After the data assembly and gene prediction, the final non-redundant gene set contained 3,024,491 ORFs, with an average length of 517 bp, and 32.28% of the ORFs appeared complete (Table S1). Based on known sequences from the NCBI NR database, 1,596,042 genes (90.72%) from all samples could be assigned to the kingdom-level taxa, in which “Bacteria” was the most abundant microbial populations. We could annotate 86.68% of the total number of genes to the phylum level, and *Firmicutes* was identified as the most dominant taxa in the SCBs bioaerosol microbiome, followed by *Bacteroidetes*, *Actinobacteria*, and *Proteobacteria*. Additionally, 60.48% of genes from all samples could be classified at the genus level and 89.20% of those could be annotated to the species level (53.95% of the total number of genes in the SCBs bioaerosol catalog) (Figure 2).

**Figure 3:**
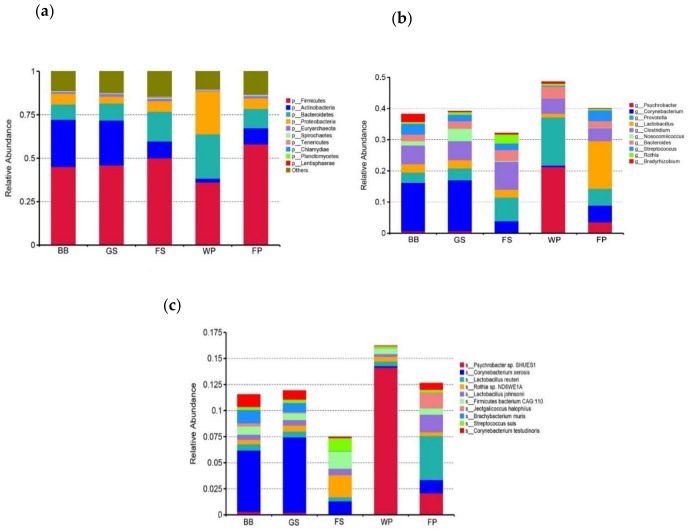
The relative abundances of predominant taxa at the phylum (**a**), genus (**b**), and species (**c**) level, among all samples. BB, GS, FS, WP, and FP refer to the bioaerosols collected from the piggeries that exclusively housed breeding boars, gestating sows, farrowing sows, weaning piglets, and finishing pigs, respectively.

For the five bioaerosol samples, “Bacteria” predominated with relative abundance level of 91.95%, 90.43%, 88.81%, 93.36%, and 90.69% in the BB, GS, FS, WP, and FP bioaerosols, respectively. Bioaerosol from WP exhibited a lower abundance of “Archaea”, 0.47%, compared to bioaerosols from other kinds of SCBs (more than 1.1%) (Figure S1a). The top 20 genera present in all samples were identified and *Prevotella* and *Clostridium* were the most abundant taxa, with a relative abundance of 6.77% and 6.62%, respectively (Figure S1b). Taxonomical assignments also indicated that the microbial composition varied between samples in each taxonomy hierarchy, particularly for the phylum, genus, and species. In comparison, the most abundant phyla were *Firmicutes* (44.99% in BB bioaerosol, 45.98% in GS bioaerosol, 50.04% in FS bioaerosol, 36.15% in WP bioaerosol, and 58.06% in FP bioaerosol), followed by *Actinobacteria* (27.23% in BB bioaerosol, 25.85% in GS bioaerosol, 9.71% in FS bioaerosol, 2.24% in WP bioaerosol, and 9.32% in FP bioaerosol), *Bacteroidetes* (8.81% in BB bioaerosol, 9.78% in GS bioaerosol, 17.04% in FS bioaerosol, 25.49% in WP bioaerosol, and 11.11% in FP bioaerosol), and *Proteobacteria* (6.16% in BB bioaerosol, 3.96% in GS bioaerosol, 6.17% in FS bioaerosol, 24.69% in WP bioaerosol, and 6.14% in FP bioaerosol) (Figure 3a). At the genus level, *Corynebacterium* was the most dominant taxon in both BB and GS bioaerosols. In contrast, *Clostridium*, *Psychrobacter*, and *Lactobacillus* were the most abundant genera in the FS, WP, and FP bioaerosols, respectively (Figure 3b). At the species level, *Corynebacterium xerosis* was the most abundant taxon in both BB and GS bioaerosols and the *Rothia* sp. *ND6WE1A*, *Psychrobacter* sp. *SHUES1*, and *Lactobacillus reuteri* were the most abundant species in the FS, WP, and FP bioaerosols, respectively (Figure 3c).

**Figure 4:**
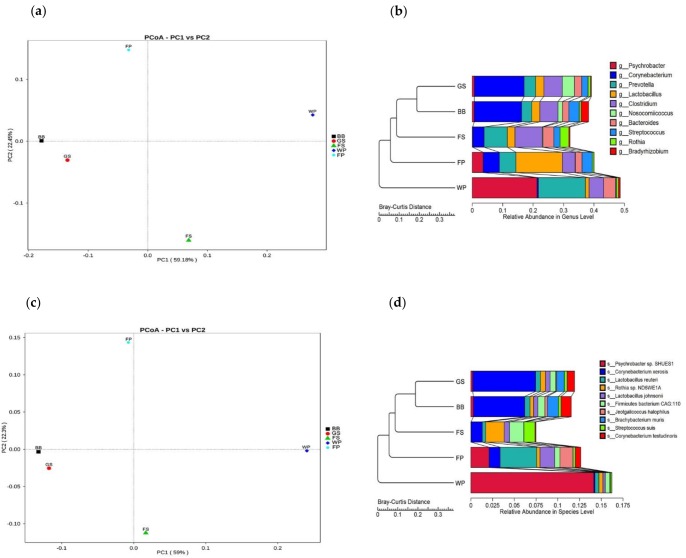
Principle coordinate analysis (PCoA) and cluster analysis of the bioaerosol microbiome from different types of swine confinement buildings. (**a**) PCoA and (**b**) clustering plots, based on the Bray Curtis dissimilarity of the detected genera. (**c**) PCoA and (**d**) clustering plots, based on the Bray Curtis dissimilarity of the detected species. BB, GS, FS, WP, and FP refers to the bioaerosols collected from exclusively from piggeries housing breeding boars, gestating sows, farrowing sows, weaning piglets and finishing pigs, respectively.

Principle coordinate analyses of all bioaerosol samples, based on the Bray Curtis dissimilarity of the detected species and genera showed that the bioaerosol microbiota of GS shared a higher similarity with that of BB, than those from the FS, WP, and FP (Figure 4a,c). Cluster analyses based on the Bray Curtis dissimilarity of detected species and genera, also confirmed that the GS bioaerosol microbiota was more similar to the BB than the other SCBs categories (Figure 4b,d).

### 3.2. Functional Characterization of the Metagenomic Profile

**Figure 5:**
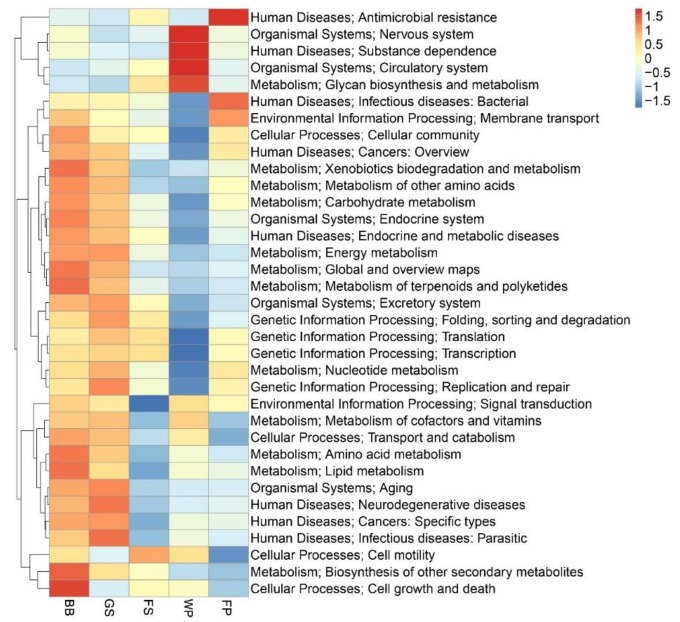
Annotated functional profile (KEGG level 2) of bioaerosols from five types of swine confinement buildings. BB, GS, FS, WP, and FP referred to bioaerosols collected from the piggeries that were exclusively housing breeding boars, gestating sows, farrowing sows, weaning piglets, and finishing pigs, respectively.

Annotating the bioaerosol gene sets, using the KEGG and eggNOG databases revealed that most functions belonged to pathways involved in metabolism, replication, recombination, and repair (Figure S2). It was apparent that most detected functions were shared across all bioaerosols, suggesting potential similarities in metabolic capabilities of the bioaerosol microbiome from the different types of SCBs. However, the heatmap displaying the abundances of the top 35 pathways in the KEGG level 2, showed more similarity between the BB and GS bioaerosols (Figure 5). These two bioaerosol samples also displayed a greater similarity in the overall functional profile, as indicated by a smaller Bray Curtis dissimilarity of the assigned KEGG and eggNOG orthologous groups (Figure S3). The heatmap displaying the abundances of the eggNOG functional classes showed that the BB and GS bioaerosols had more genes related to RNA processing and modification and secondary metabolites metabolism, than the other types of SCBs. The abundances of genes involved in extracellular structures, cell cycle control, cell motility, signal transduction mechanisms, cell wall biogenesis, posttranslational modification, and protein turnover were the highest in the WP bioaerosol than the other types of SCBs. Conversely, genes assigned to carbohydrate transport and metabolism and ribosomal structure and biogenesis were less abundant in the WP bioaerosol than other types of SCBs. Moreover, genes related to the cytoskeleton were the most abundant in the FS bioaerosol than the other types of SCBs (Figure S4).

### 3.3. Occurrence, Abundance, and Diversity of ARGs

**Figure 6:**
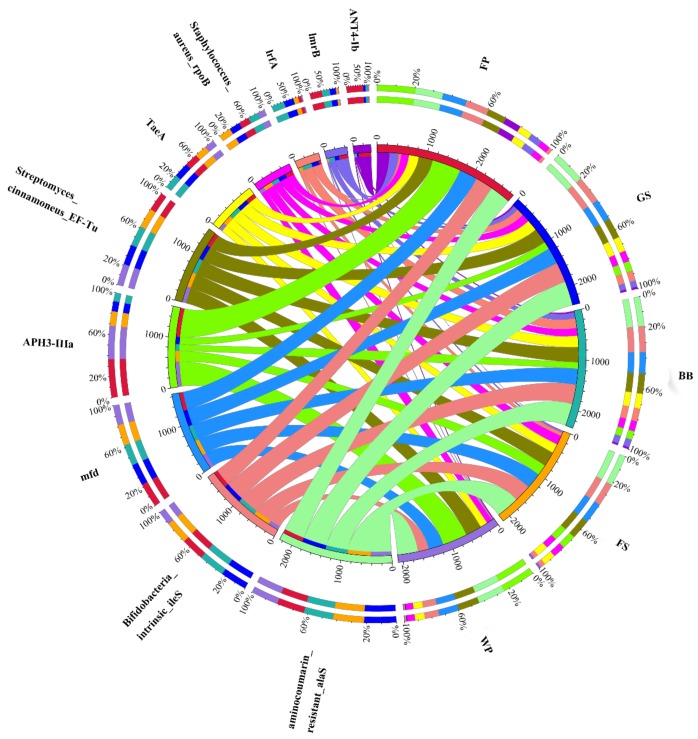
Distribution of the top 10 ARGs subtypes detected in all samples. Data were visualized using Circos. The two outermost circles list the names of the samples and each ARG subtype. The third circle represents the gene number of the ARG subtype. The width of the bars between the ARG subtypes and the samples correlate to the percentage of corresponding ARG subtype in the samples. The outset cycles were colored according to the software default settings. BB, GS, FS, WP, and FP refer to the bioaerosols collected from piggeries that were exclusively housing breeding boars, gestating sows, farrowing sows, weaning piglets, and finishing pigs, respectively.

To explore the profile of ARGs present in bioaerosols, we conducted a BLASTP analysis of the bioaerosol microbiome against the ARDB and showed that the BB, GS, FS, WP, and the FP bioaerosols had 63,400, 60,337, 51,528, 59,511, and 73,254 ARGs, respectively, which could be assigned to 304, 300, 300, 277, and 304, known as the ARG subtypes, respectively (Figure S5). The top 10 ARG subtypes in all samples contained genes conferring resistance to aminoglycosides, aminocoumarin, mupirocin, elfamycin, fluoroquinolone, pleuromutilin, rifampin, and lincosamide (Figure 6). Heatmap displaying the abundances of the top 30 ARGs subtypes in individual samples showed that the most dominant ARGs subtype in the BB, GS, and FS bioaerosols was Aminocoumarin_resistant_alaS (aminocoumarin resistance gene, with the relative abundance of 11.24%, 13.10%, and 14.82%, respectively), while the most dominant ARGs subtype in, both, the WP and FP bioaerosols was APH3-IIIa (aminoglycoside resistance gene, with the relative abundance of 13.72% and 12.38%, respectively). Moreover, the clustering of samples based on the ARG subtype profile showed that the ARG subtypes composition of the BB, GS, and FS shared a high similarity, while the WP and FP were more similar (Figure S6).

### 3.4. Co-Occurrence Pattern between ARGs and Microbial Community

**Figure 7:**
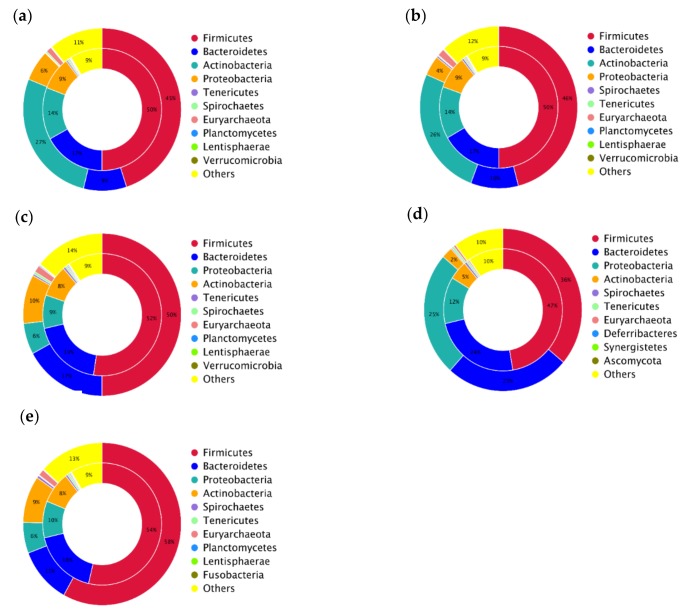
The attribution analysis of ARGs and the bacteria species in BB (**a**), GS (**b**), FS (**c**), WP (**d**), and FP (**e**) bioaerosols are showed by the circle maps. The inner circle shows the distribution of the bacteria species of the total ARGs, and the outer circle shows the species distribution of all samples. BB, GS, FS, WP, and FP refer to the bioaerosols collected from the piggeries that were exclusively housing breeding boars, gestating sows, farrowing sows, weaning piglets, and finishing pigs, respectively.

WMS can obtain information about the microbial composition and ARGs profile of the microbiome at the same time, we thus, investigated the co-occurrence pattern between the microbial composition and the ARG profiles in the bioaerosol microbiomes. *Firmicutes* and *Bacteroidetes* were the two phyla that carried far more ARGs than the other phyla. *Firmicutes* carried 50%, 50%, 52%, 47%, and 54% of the total antibiotic resistance genes in the BB, GS, FS, WP, and FP bioaerosol microbiomes, respectively. *Bacteroidetes* carried 17%, 17%, 19%, 24%, and 18% of the total antibiotic resistance genes in the bioaerosol communities of BB, GS, FS, WP, and FP, respectively. For *Firmicutes* and *Bacteroidetes*, the percentages of carried ARGs were higher than their proportions in the microbial communities, indicating that both *Firmicutes* and *Bacteroidetes* were carrying more resistance genes than the other phyla. *Actinobacteria* predominated the bioaerosol microbiome of the BB, GS, and WP, with the relative abundance of 27%, 26%, and 25%, respectively. However, the percentages of carried ARGs of *Actinobacteria* were lower than the abundances of phylum *Actinobacteria*, in these three communities, indicating that the *Actinobacteria* carried fewer resistance genes than the other phyla. Additionally, *Proteobacteria* in all bioaerosol microbiomes also carried some resistance genes (Figure 7).

## 4. Discussion

In the modern large-scale pig production, animals with high densities raised in the confinement buildings, resulted in a poor indoor air quality [1]. SCBs bioaerosols can be emitted into the external environment and, therefore, can cause harm to public health; the microorganisms within the SCB bioaerosols are the main contributors to the adverse effects of bioaerosols on human health [2,3,4]. The production phase has been recognized as an important factor influencing the taxonomical profile of SCB bioaerosols [2], but the differences in functional capabilities and ARG profiles of bioaerosols between the piggery types, sorted by the production phase remains, to be understood. Therefore, the present study compared the microbial taxonomical, functional potential, and ARG profiles of bioaerosols from different types of buildings, which exclusively housed weaning piglets, finishing pigs, gestating sows, farrowing sows, and breeding boars, respectively, to determine whether the production phase would affect the bioaerosol microbiomes of the SCBs.

Bacteria has been shown to predominate the bioaerosol microbiota of SCBs, whereas the abundances of fungi and archaea were extreme low [32]. The present study also showed that the abundance of bacteria was far more than that of other taxa, at the kingdom level, in the SCB bioaerosols. In addition, the predominant phyla in the SCBs bioaerosols were *Firmicutes*, *Bacteroidetes*, *Actinobacteria*, and *Proteobacteria*, which were also predominant in the atmospheric environment of other places, like urban hospital or office [2,33], suggesting that the species belonging to these four phyla are the main components of airborne microbial communities. A previous study showed that the production phase could affect the taxa abundances at the phylum level [2]. In the present study, the abundance of *Actinobacteria* in the BB and FS bioaerosols was higher than that in other SCB bioaerosols, which might be associated with the higher abundance of *Actinobacteria*, in the feces of breeding boars and suckling piglets [10,34]. Moreover, we found that the WP bioaerosol had higher abundances of *Bacteroidetes* and *Proteobacteria*, as well as lower abundances of *Firmicutes* and *Actinobacteria* than the other SCBs bioaerosols. Previous studies that compared the fecal microbiota composition between pigs at different growth stages, revealed that the ratio of *Bacteroidetes* to *Firmicutes* and the abundance of *Proteobacteria* in weaning piglets, were significantly higher than those in older pigs. Furthermore, fecal *Actinobacteria* abundance increased along with the gained weight [9,35]. These results suggest that the pattern of the production phase influencing the SCB bioaerosol communities is similar to the succession of swine fecal microbiota, over time. The predominant genera *Prevotella*, *Clostridium*, and *Bacteroides* in the SCBs bioaerosols were found to be commonly associated with the intestinal tract of pigs, further supporting the hypothesis that swine feces are the major source of the bioaerosol microorganisms in SCBs [2,8,16]. Previous studies have showed that WP and FP bioaerosols have a higher abundance of *Prevotella* than the GS and FS bioaerosols [2]. In this study, a higher abundance of *Prevotella* was observed in the WP bioaerosol, compared to the other SCBs bioaerosols, which might be attributed to the gradual decline of fecal *Prevotella* abundance with an increased swine age [11]. *Lactobacillus* has been shown to increase as the pigs aged [10,36,37], we also got similar results showing that the FP bioaerosol has a higher abundance of *Lactobacillus*, compared to the WP bioaerosol. A previous study showed that the abundance of *Lactobacillus* was higher in castrated pigs than female pigs and breeding boars [34]. However, we didn’t detect any difference in *Lactobacillus* abundance between the BB and GS bioaerosols. Interestingly, we found that the FP bioaerosol exhibited the highest abundance of *Lactobacillus* than other SCBs bioaerosols, which was comparable to the previous findings showing *Lactobacillus* to be more abundant in finishing pigs than in weaning piglets and sows [32]. Moreover, we found that *Psychrobacter* was more abundant in the WP bioaerosol. *Psychrobacter* is a genus of the Gram-negative bacteria, which is known to be a kind of facultative psychrophiles that is able to grow at a large temperature range [38]. The temperature in the WP was higher than the other SCB categories, so the reason behind the WP bioaerosol exhibiting the highest abundance of *Psychrobacter* needs to be addressed in future studies. *Corynebacterium* has been shown to predominate the bioaerosol microbiota in the FP and FS buildings [39]. However, a higher abundance of *Corynebacterium* was observed in BB and GS bioaerosols, in the present study. This bias might be attributed to the different methods for sampling and assessing the microbial community used in the present study, than in the previous study. Additionally, the most abundant taxa at the species level differed between the SCBs categories, which provided new insights into the effects that the production phase had on the bioaerosol communities. In agreement with previous findings [2], we also found that the bioaerosol communities could be sorted by the production phase, based on the Bray Curtis dissimilarity, indicating that the production phase exerts a significant influence on the microbial composition in the SCBs bioaerosols. BB and GS shared a higher similarity in the taxonomical profile than the other piggery types, which might be attributed to the interactions between these two types of piggeries, since the boars were frequently used to stimulate the onset of oestrus, in the weaning sows, which were also kept in the GS. However, the present study only sequenced one pooled sample for each production phase, further studies, testing more samples rather than a pooled sample within each piggery type, are needed, to compare the variances across samples.

In the KEGG analysis, genes associated with carbohydrate and amino acid metabolism were enriched in SCBs bioaerosols, which was similar to the functional potential of airborne microbiome from other environments, and the swine fecal microbiome [40,41]. Beyond the microbial composition, the bioaerosol microbiomes also differed between the SCB categories, in terms of functional potential. The higher abundances of genes associated with protein turnover were observed in the WP bioaerosol microbiome, which might be attributed to the increased use of amino acid for protein accretion and consumption of high protein diet, in weaning piglets [41]. Likewise, owing to the lower level of complex polysaccharides in the weaning diet, the lower abundances of genes associated with carbohydrate transport and metabolism were found in the WP bioaerosol microbiome [41]. Therefore, the WP bioaerosol exhibited a higher protein turnover capability and lower carbohydrate utilization ability than others. Studying the SCB bioaerosol metagenome also sheds light on the antibiotic-resistant genes employed by the microbiome. Since antibiotics were widely used as the growth promoter in feed or water, within swine feeding operations, most metagenomic sequences retrieved from the swine fecal metagenome were found to be involved in the antibiotic resistance mechanisms [41,42]. In the present study, the higher occurrence and diversity of ARGs was observed in the FP bioaerosol microbiome, which might be attributed to a longer exposure of the finishing pigs to antibiotics, for meat production [43]. Aminocoumarin resistance gene was found to predominate the microbiomes of the BB, GS, and FS bioaerosols, and might be explained by the increased therapeutic use of aminocoumarin in boars, gestating sows, and farrowing sows. Novobiocin, a main member of aminocoumarin, was extensively used to treat boars and sows infected with salmonella [44]. Interestingly, there was no history of novobiocin use on the swine farm, which was the sampling site of the present study, indicating that the aminocoumarin resistance gene might have been derived from other farms. Aminoglycoside antibiotics, such as gentamicin, neomycin and streptomycin, were frequently used to manage post-weaning diarrhea of pigs [45]. In the present study, we observed a higher abundance of aminoglycoside resistance gene in the WP bioaerosol, which might be associated with the use of aminoglycoside antibiotics on the weaning piglets. In the large-scale farm, weaning piglets were transferred into the FP, at the end of the nursery period, and it was possible that the importation of the weaning piglets into the FP resulted in highly similar ARGs profiles between the WP and FP bioaerosols [2]. Consistently, we also observed a high similarity in the ARGs composition between the WP and FP bioaerosols. Moreover, we found a high similarity in the ARGs composition, among the BB, GS, and FS bioaerosols, which is likely due to the proximity and interaction between these three production phases. Previous studies have showed that the occurrence of ARGs was correlated with the microbial composition [46,47]. In the present study, *Firmicutes* and *Bacteroidetes* carried more antibiotic resistance genes than the other taxa, which is consistent with previous studies showing that higher number of ARGs are found in *Bacteroidetes* [47]. Collectively, FP had a higher ARGs occurrence and diversity than others, and the most dominant ARG in bioaerosol, differed between the piggery types.

## 5. Conclusions

Our study is the first to apply the whole metagenome shotgun sequencing technique to characterize the composition and functional potential of the bioaerosol microbial populations in the swine confinement buildings from different production phases. The data from this study suggest that the production phase exerts an effect on the microbial composition, functional potential, and the ARG profile of the SCB bioaerosol microbiome. High similarity in the bioaerosol microbiome is presented in buildings that housed breeding boars and gestating sows.

## Supplementary Materials

The following are available online at https://www.mdpi.com/2076-2615/9/3/90/s1, Figure S1: The relative abundances of taxa at the domain level among all samples (a) and the top 20 genera in the bioaerosol microbiome (b). BB, GS, FS, WP, and FP refer to the bioaerosols collected from the piggeries that were exclusively housing the breeding boars, gestating sows, farrowing sows, weaning piglets, and finishing pigs, respectively, Figure S2: The predicted genes of bioaerosol microbiome mapped onto the KEGG pathway annotation and eggNOG functional annotation, Figure S3: PCoA of the bioaerosol samples based on functional annotation of the genes by KEGG pathway (a) and the eggNOG (b). Plots were based on the Bray Curtis dissimilarity of the assigned KEGG orthologous groups (KOs) and the eggNOG orthologous groups (NOs). BB, GS, FS, WP, and FP refer to the bioaerosols collected from the piggeries that were exclusively housing the breeding boars, gestating sows, farrowing sows, weaning piglets, and finishing pigs, respectively, Figure S4: Annotated functional profile (eggNOG level 1) of the bioaerosols from the five types of swine confinement buildings. BB, GS, FS, WP, and FP refer to the bioaerosols collected from the piggeries exclusively housing the breeding boars, gestating sows, farrowing sows, weaning piglets, and the finishing pigs, respectively, Figure S5: Total number of antibiotic resistant genes (ARG) (a) and ARG subtypes (b) detected in the bioaerosol samples. BB, GS, FS, WP, and FP refer to the bioaerosols collected from the piggeries that were exclusively housing the breeding boars, gestating sows, farrowing sows, the weaning piglets, and the finishing pigs, respectively, Figure S6: Heatmap of the ARG subtype abundances in all samples. BB, GS, FS, WP, and FP refer to the bioaerosols collected from the piggeries that were exclusively housing the breeding boars, gestating sows, farrowing sows, weaning piglets and finishing pigs, respectively, Table S1: Overall gene catalogue profile.
