# Supplementary material for: Production Phase Affects the Bioaerosol Microbial Composition and Functional Potential in Swine Confinement Buildings
Source: Animals (Basel). 2019 Mar 12;9(3):90. doi: 10.3390/ani9030090 (PMC6466638; doi:10.3390/ani9030090)
Supplement: Supplementary file 1 [file animals-09-00090-s001.pdf]

Supporting information

Production phase affects the bioaerosol microbial composition and functional potential in swine confinement buildings

Honglin Yan [1,2], Li Zhang [1], Zhendong Guo [3], Hongfu Zhang [2], Jingbo Liu [1,2,*]

[1] School of Life Science and Engineering, Southwest University of Science and Technology, Mianyang 621010, China
[2] State Key Laboratory of Animal Nutrition, Institute of Animal Sciences, Chinese Academy of Agricultural Sciences, Beijing 100000, China
[3] Key Labortatory of Jilin Province for Zoonosis Prevention and Control, Veterinary Research Institute, Chinese Academy of Agriculture Sciences, Changchun 130122, China
* Correspondence: liuswust@163.com; Tel: +86-0816-6089521 (J.B.Liu)

Table S1. Overall gene catalogue profile

**Gene prediction**	
ORFs number	3,024,491
Average length (bp)	517.66
Complete ORFs percentage (%)	32.28
GC percentage (%)	48.56

a

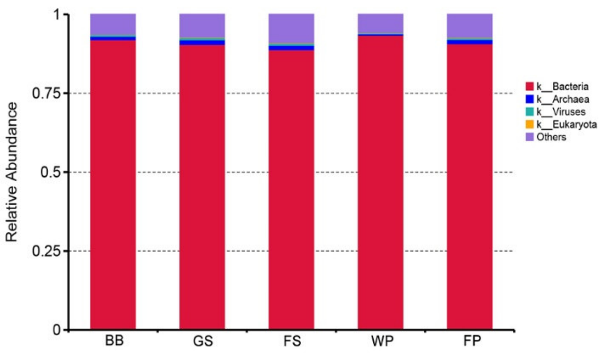

b

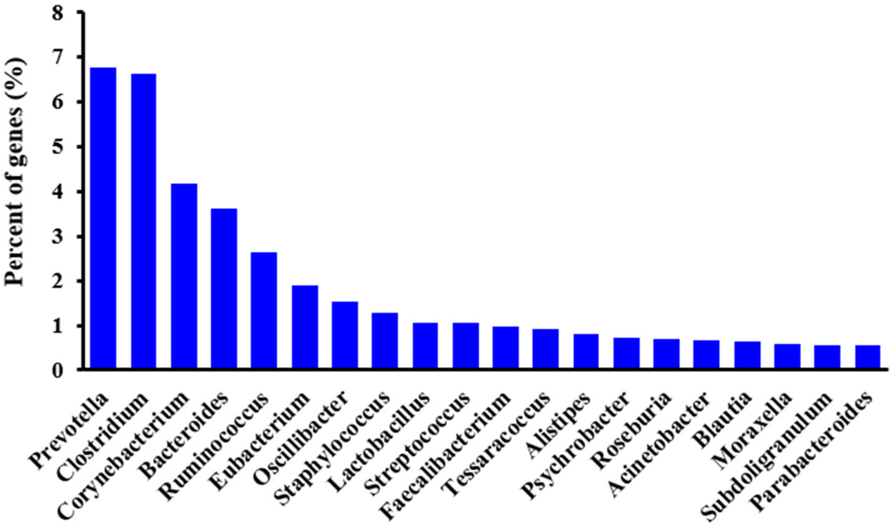

Figure S1. The relative abundances of taxa at the domain level among all samples (a) and the top 20 genera in bioaerosol microbiome (b). BB, GS, FS, WP and FP refer to the bioaerosols collected from the piggeries that were exclusively housing the breeding boars, gestating sows, farrowing sows, weaning piglets, and finishing pigs, respectively.

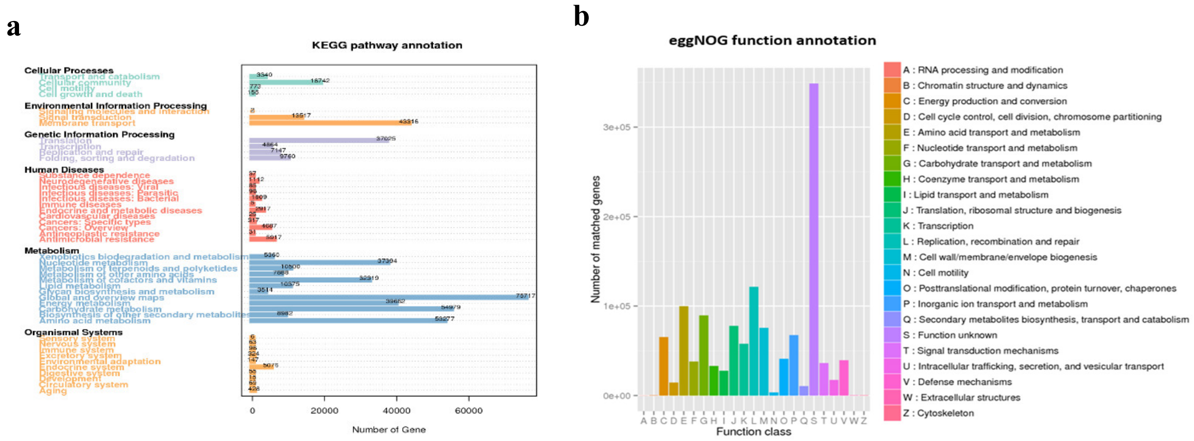

Figure S2. The predicted genes of bioaerosol microbiome mapped onto the KEGG pathway annotation and eggNOG functional annotation.

a b

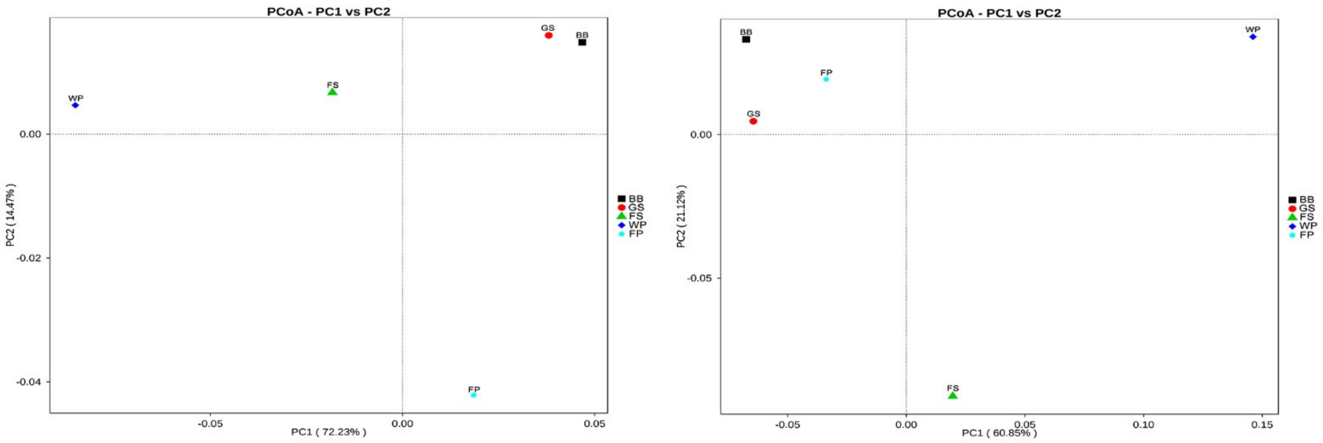

Figure S3. PCoA of bioaerosol samples based on functional annotation of the genes by KEGG pathway (a) and eggNOG (b). Plots were based on Bray Curtis dissimilarity of the assigned KEGG orthologous groups (KOs) and the eggNOG orthologous groups (NOs). BB, GS, FS, WP and FP refer to bioaerosols collected from the piggeries that were exclusively housing breeding boars, gestating sows, farrowing sows, weaning piglets and finishing pigs, respectively.

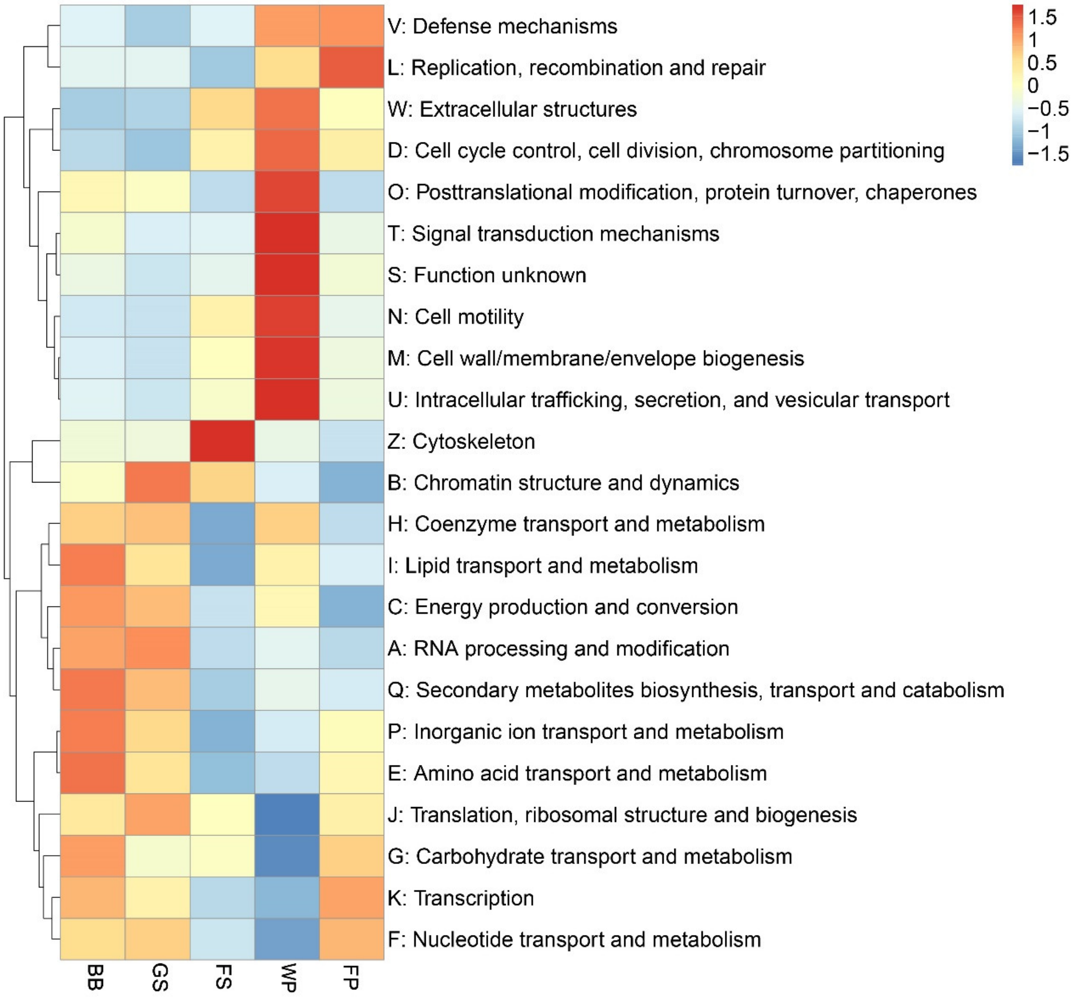

Figure S4. Annotated functional profile (eggNOG level 1) of the bioaerosols from the five types of swine confinement buildings. BB, GS, FS, WP and FP refer to bioaerosols collected from the piggeries that were exclusively housing breeding boars, gestating sows, farrowing sows, weaning piglets and finishing pigs, respectively.

a

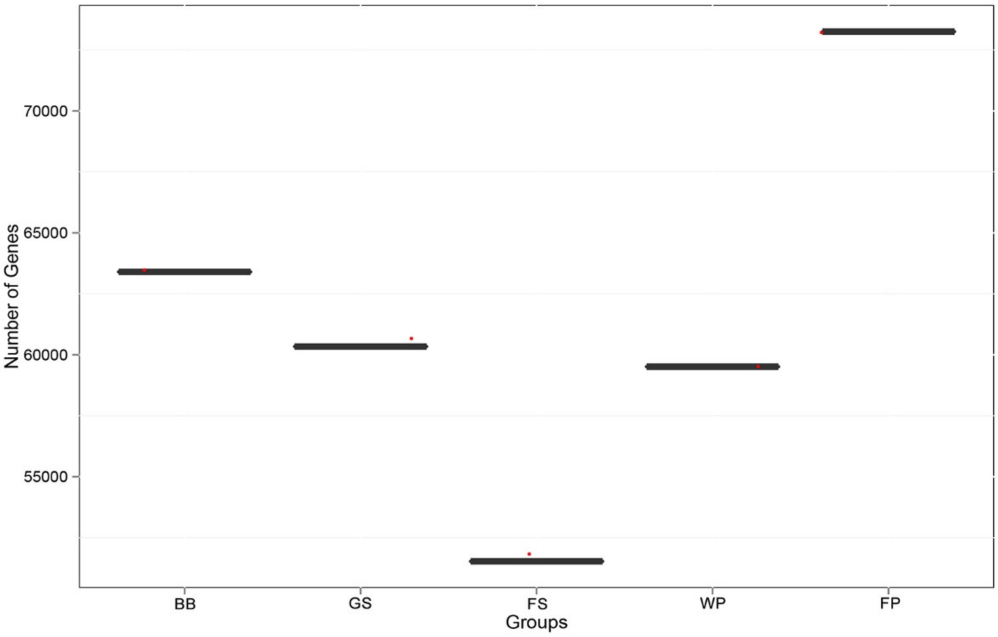

b

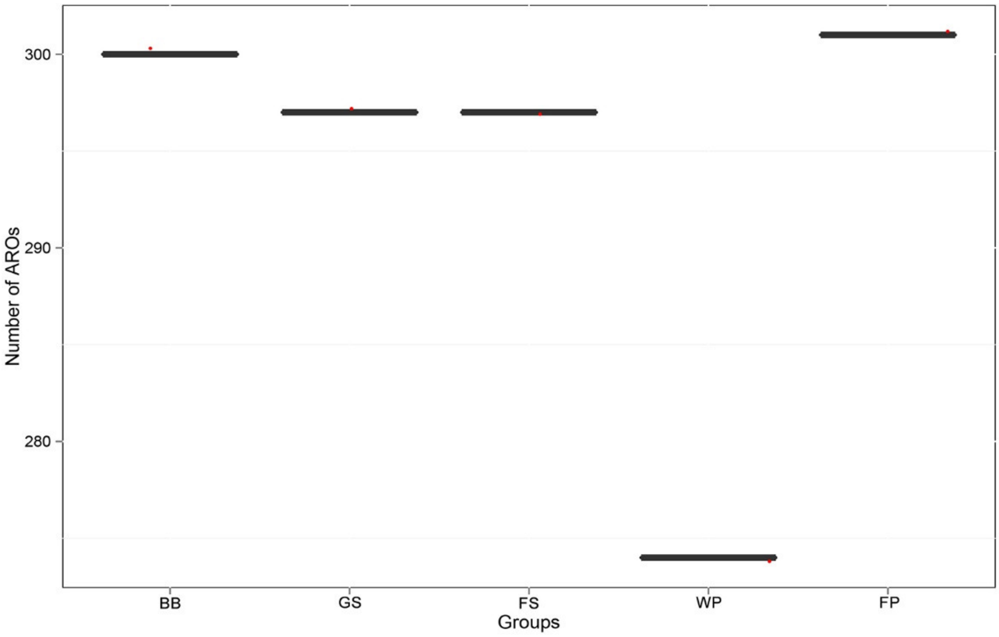

Figure S5. Total number of antibiotic resistant genes (ARG) (a) and ARG subtypes (b) detected in bioaerosol samples. BB, GS, FS, WP and FP refer to bioaerosols collected from the piggeries that were exclusively housing breeding boars, gestating sows, farrowing sows, weaning piglets and finishing pigs, respectively.

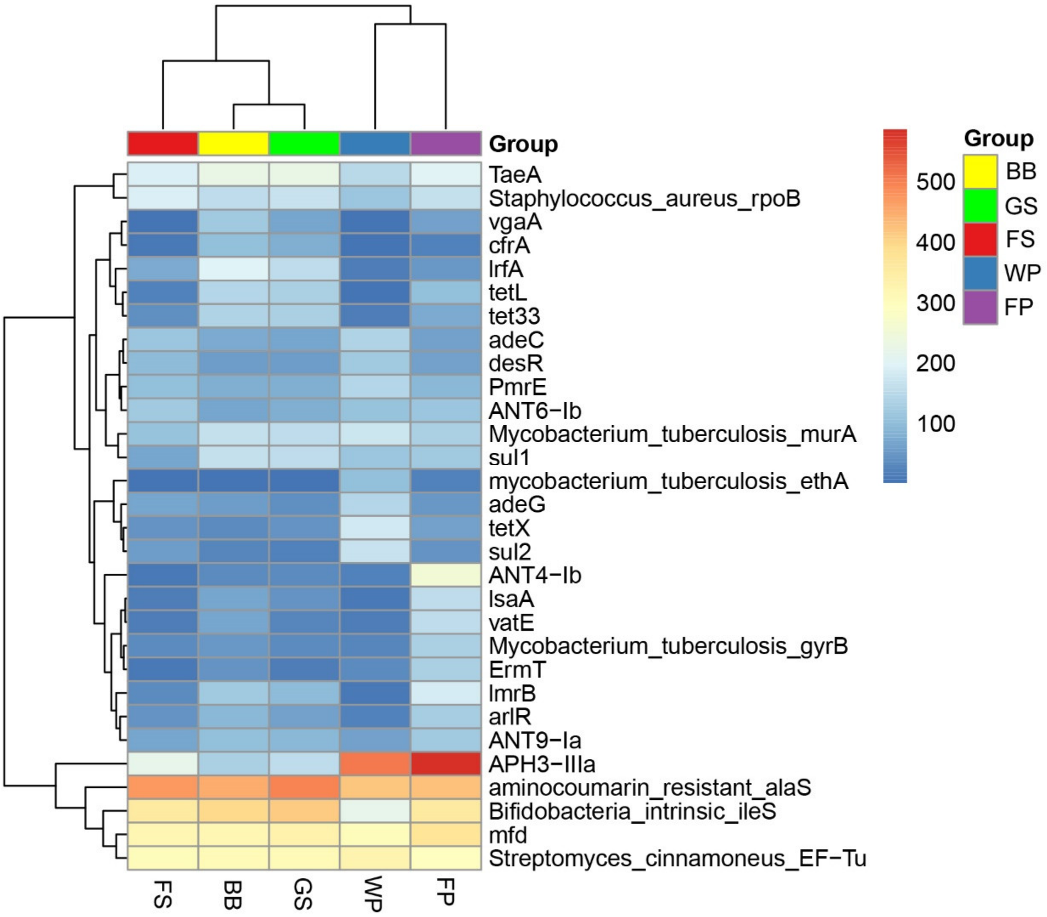

Figure S6. Heatmap of ARG subtype abundances in all samples. BB, GS, FS, WP and FP refer to bioaerosols collected from the piggeries that were exclusively housing breeding boars, gestating sows, farrowing sows, weaning piglets and finishing pigs, respectively.
